# Incident vertebral fractures 12 months following glucocorticoid initiation in children with rheumatic disorders

**DOI:** 10.1186/1546-0096-10-S1-A75

**Published:** 2012-07-13

**Authors:** Bianca A Lang, Celia Rodd, Timothy Ramsay, David A Cabral, Peter B Dent, Janet E Ellsworth, Kristin M Houghton, Adam Huber, Roman Jurencak, Maggie Larché, Claire MA LeBlanc, Brian Lentle, MaryAnn Matzinger, Paivi M Miettunen, Kiem Oen, Johannes Roth, Claire Saint-Cyr, Rosie Scuccimarri, Nazih Shenouda, Leanne M Ward

**Affiliations:** 1 Dalhousie University, Halifax, NS, Canada; 2 McGill University, Montreal, QC, Canada; 3 McMaster University, Hamilton, ON, Canada; 4 National Pediatric Bone Health Working Group, Ottawa, ON, Canada; 5 Université de Montréal, Montreal, QC, Canada; 6 University of Alberta, Edmonton, AB, Canada; 7 University of British Columbia, Vancouver, BC, Canada; 8 University of Calgary, Calgary, AB, Canada; 9 University of Manitoba, Winnipeg, MB, Canada; 10 University of Ottawa, Ottawa, ON, Canada

## Purpose

Compromised bone health is recognized as an important source of morbidity among children with glucocorticoid (GC)-treated rheumatic diseases (RD). The aims of this study were to determine the frequency and characteristics of incident vertebral fractures (VF) 12 months after GC initiation in a prospectively-followed cohort of children with RD, and to examine clinical factors associated with their development.

## Methods

Children (< 17 years) initiating GC for treatment of RD between January 2005 and December 2007 in ten participating Canadian tertiary pediatric centers were enrolled in the Steroid-Associated Osteoporosis in the Pediatric Population (STOPP) study. Enrolled patients had baseline (within 30 days of initiating GC) and 6- monthly spine areal (a) BMD studies. Additionally, radiographs of the thoracolumbar spine at baseline and 12 months (m) were evaluated using the Genant semi-quantitative method. Fractures present at baseline (prevalent VFs) were documented. An incident VF was defined as a new VF or worsening of an existing fracture. Patients also had baseline and 3 monthly assessment of GC exposure, clinical status including disease activity (measured on a 10 cm visual analogue scale by the patient’s rheumatologist), and questionnaires to determine physical activity and vitamin D/calcium intake.

## Results

Of 135 patients enrolled, data were available on 118 (64% female, median age 10.8 years) at 12 m. Diagnoses included juvenile dermatomyositis (JDM) (23%), juvenile idiopathic arthritis (JIA) (36 %), systemic lupus (SLE) and related conditions (18%), systemic vasculitis (14%), and other ( 9%). At 12 m, 7 patients (6%) had 12 incident VFs (3 SLE, 2 JDM, 1 vasculitis, 1 overlap). All incident VFs were new fractures; 5 patients had a single VF, one had 2 VFs and one had 5 VFs. Three patients had mild and 4 had moderate VFs. Nine (75%) of the incident VFs were thoracic and 11 (92%) had wedge morphology. Patients with and without incident VFs were similar for age, gender, pubertal status, disease activity, physical activity, vitamin D/calcium intake and presence of back pain. The decrease in spine aBMD and increase in BMI in the first 6 months was larger in those with incident VFs (Δ spine aBMD Z-score mean -0.8, SD 0.4; Δ BMI Z-score +1.7, SD 1.0) versus those without (Δ spine aBMD Z-score -0.4, SD 0.5; Δ BMI Z-score +0.5 SD 0.8). Cumulative GC dose (g/m^2^) was almost double in those with incident VFs (median 9.3 g/m^2^, range 3.0-12.5) versus those without (median 5.4, range 0.01-27.5); with the greatest difference in GC dose seen within the first 6m. None of the 9 children with baseline prevalent VFs had incident fractures. In 8 of these 9 patients, spine status had improved overall at 12 m.

## Conclusion

Incident VFs occurred in a small proportion of our cohort (6%); however one patient was more severely affected. Children with incident VFs received almost twice as much GC therapy as those without, had a greater decline in spine BMD, and manifested greater increases in BMI. Back pain and baseline prevalent VFs did not appear to be associated with incident VFs. Funded by CIHR.

## Disclosure

Bianca A. Lang: None; Celia Rodd: None; Timothy Ramsay: None; David A. Cabral: None; Peter B. Dent: Roche , 6; Janet E. Ellsworth: None; Kristin M. Houghton: None; Adam Huber: None; Roman Jurencak: None; Maggie Larché: None; Claire M.A. LeBlanc: None; Brian Lentle: None; MaryAnn Matzinger: None; Paivi M. Miettunen: None; Kiem Oen: None; Johannes Roth: None; Claire Saint-Cyr: None; Rosie Scuccimarri: None; Nazih Shenouda: None; Leanne M. Ward: None; and the Canadian STOPP Consortium: None.

